# A Qualitative Content Analysis of Online Public Mental Health Resources for COVID-19

**DOI:** 10.3389/fpsyt.2022.553158

**Published:** 2022-02-24

**Authors:** Faith Martin, Thomas Oliver

**Affiliations:** ^1^Centre for Intelligent Healthcare, Coventry University, Coventry, United Kingdom; ^2^Department of Social Sciences, University of the West of England, Bristol, United Kingdom

**Keywords:** information quality, online intervention, qualitative, mental health, COVID-19, mass intervention, online health information

## Abstract

The COVID-19 pandemic has far reaching potential public mental health impacts and is linked to higher levels of depression and anxiety. To address these in part, online information resources acted as mass interventions. It is vital to explore the content of these interventions, to consider the framing of the pandemic and to examine the extent to which their content is relevant. In March 2020, a qualitative content analysis was undertaken of 39 easily accessible online resources that offered advice, tips or guidance relating to mental health or mental wellbeing and COVID-19. Their content was compared to subsequent reports of the mental health impact of the pandemic. Resources frequently focused on anxiety. The content of intervention was typically of a cognitive-behavioral nature, with a significant focus on maintaining social contact. Typically, distress related to the situation was normalized and stigmatizing language was not seen. Data revealed a significant impact of the pandemic on depression as well as anxiety measures in the general UK population. A key recommendation is to ensure both depression and anxiety are addressed in these public mental health resources.

## Introduction

The COVID-19 pandemic presented a major public health and public mental health challenge. At the start of the pandemic in March 2020, reviews of the impact of restrictions such as quarantine and COVID-19 itself pointed to an understandable effect on psychological wellbeing and mental health. Prior to COVID-19, research on the psychological impacts of epidemics and quarantine suggested high risk of potential distress including confusion, loss, anger, frustration, low mood, fear, stress, insomnia and boredom, with a minority of participants reporting high levels of depressive symptoms and anxiety (1–7). Given the impact on entire populations, often under protective measures that restrict their movement and interaction with others, even a minority of the population represents a significant number of people in distress and a significant challenge to the mental health services. UK data suggest that anxiety and depression levels “spiked” at the point at which the lockdown strategy was announced (8), prompting online public mental health information.

“Lockdown” and threat of illness are stressful. Stopping work, loss of routine, reduced physical social contact, a sense of isolation and loss of day-to-day activities are all identified as stressors, which can be addressed with coping and stress management techniques, problem solving, self-care, alleviation boredom, use of social networks, engagement with reliable information and re-iterating the purpose of isolation is to keep others safe (1, 2, 9). Sharing basic strategies to manage this distress has been recommended (10), alongside a need for online psychoeducation and self-management support, with self-directed interventions (7, 11–13).

Numerous online resources offering support and guidance on how to manage one's mental health were published in March 2020, in the period where the pandemic rose to the public's awareness and protective measures were enacted in many nations. These were effectively mass interventions, to attempt to maintain psychological wellbeing or minimize adverse impact on mental health. Many online resources were produced, however for these to be useful, their content must be optimized. Some would argue that anxiety and distress in the face of this global health threat is entirely normal. However, it is also important to offer support and strategies to maintain wellbeing, and further intervention where people require more specialist mental health support.

The content of online resources intending to address mental health needs to be explored, to highlight any areas of omission in particular. This is important both at the time of the protective measures being put into place and subsequently, to learn from this pandemic and plan for the next. Indeed, future pandemics are thought to be “inevitable” (14). Mental health is key in their management: avoiding or treating depression and anxiety is important in its own right, but also as they may be linked to lower engagement in adaptive health-behaviors needed for COVID-19, as they are for many other health behaviors (15). The way in which these resources frame both COVID-19 and mental health is also important to understand and potentially improve. Excessive fear arousal or global descriptions of lack of personal control are likely to risk both mental health and adherence to public health infection reduction measures (16, 17). Stigmatizing messages must be avoided, for both mental health and COVID-19 (1). This study aimed to complete a rapid content analysis of the easily accessible, online resources to elucidate their content, comparing these to subsequent data on the mental health impact of the pandemic.

## Method

### Sampling of Resources

Google search for “COVID-19 mental health” was conducted (29/03/2020). The first twenty results that included guidance and advice were selected for analysis. An additional 10 novel results (i.e. disregarding duplicates from previous search) from a search for “COVID-19 mental wellbeing” were also selected. The searches were limited to results in English and was conducted from google.co.uk. This was augmented by Twitter searches using the same terms, selecting results not duplicating those from the Google searches. Snowballing was used to identify further resources from links within the online documentation. Links to other resources were all noted and these resources then analyzed.

Analysis began immediately with the Google search results, then Twitter results and snowballed results. Sampling was ceased when saturation was reached.

### Evidence of the Mental Health Impact of COVID-19

The sampling of resources relating to the pandemic focused was conducted in the UK, and was limited to results in English. As such, the evidence we use to compare the impact of the pandemic on mental health uses UK focused data. The Office of National Statistics (ONS) is a UK government organization that collects, analyses and reports on relevant health, wellbeing, economic and population data. During the COVID-19 pandemic, they have collected and reported data relating to anxiety and depression symptom experience in adults. Data were collected using common measures—the Patient Health Questionnaire for depression (18) and the Generalized Anxiety Disorder assessment for anxiety (19). The measures provide an indication of those with probable clinical levels of depression or anxiety. These data were used to identify the estimated actual impact on the pandemic on mental health. An ONS report on depression provides data from pre-pandemic, 2020 and January-March 2021 (20). The report on anxiety uses data from the early pandemic—from April to May 2020 (21). These are supplemented by a report presenting data from July-August 2021 (22).

### Data Analysis

The content from each resource was downloaded. Qualitative content analysis was used inductively to code the data, as this approach allows analysis of both the explicit content and the latent meaning (23). Descriptions of the (a) type of data source, (b) type of resource, and (c) focus/topics were analyzed to create discrete categories addressing each factor. The details of the guidance itself were initially described with open-ended, descriptive responses. These were inspected to form inductive categories relating to each of the six areas outlined in the data collection (key message, diagnostic categories, responsibility, mechanism for change, normalization, claims). After analysis of 10 resources, initial categories were created and each resource re-checked for the presence of all categories. As analysis continued, with each new category, the previous resources were re-checked to explore whether this category was present. All data were first analyzed by one researcher, and then second coded by the other.

Coding continued until all the initially identified resources had been analyzed. During this process, it was noted whether each new resource was adding any new data. Saturation was used as the criterion as to whether it was necessary to seek further resources. Saturation was defined as resources being redundant, in that no new data advanced the conceptual categories (24, 25). Forty percentage of the resource data were second coded, and inter-rater reliability was calculated (26), with the protocol that a kappa <0.90 would lead to second coding of all data.

## Results

The 30 resources identified from Google were supplemented with a further 19 identified from Twitter or snowballing. These included the resources published by Public Health England, UK National Health Service, World Health Organization, United States Center for Disease Control and several mental health charities. A list of the resources is given in Table 1. On close inspection, four of the resources identified from Google were excluded as they did not include novel guidance on mental health or wellbeing, and six of the resources identified from snowballing were also excluded for this reason. Analysis was conducted on 39 resources. No new themes or topics were identified at the end of the analysis of the identified resources, therefore no further sampling was conducted. Inter-rated reliability of kappa 0.94 was achieved for second coding of 40% of the resources.

**Table 1:** Lists the organizations that produced the resources.

**Source**	**Type of source**
Public health England	UK Government
Mental health foundation	Charity—public mental health
Mind	Charity—mental health
NHS Every mind matters	UK National Health Service
WHO	World Health Organsiation
Rethink mental illness	Charity for people with “Mental illness”
CDC	United States Centre for Disease Control
Mental Health UK	Charity for mental health
Mates in mind	Charity supporting mental health for employers, particularly construction industry
NI Gov	Northern Ireland Government
A24	Medical staffing business
University of London	University
Lifeline	Charity—Australian—Crisis support
Circle 2 success	Private company that supports businesses
Student minds	Student mental health charity
Truro college	College website
National survivor user network	Charity—mental health service survivors
BBC News	British Broadcasting Co-operation, journalists
Self-injury Support	Charity—mental health—self injury
NHS Every mind matters	UK National Health Service
Friendship bench	Charity—mental health—international but based in Africa
#InThisTogether	National Mental Health Commission, Australia
UCL with City Councils	University supporting a local council
Charlie waller	Charity—mental health
Psychology today	Psychology magazine
Help with mental health	For profit psychological clinical services
WCCBT	Professional confederation—World Confederation of Cognitive Behavioral Therapy
Dulwich Centre	Charity—mental health survivors—Australia
Guardian	UK newspaper
Head to health	Charity—mental health—Australia
Kevin MD	An individual's website, ran for business
PsychReg	Online psychology resource
Samaritans	Charity—mental health and crisis support
Mental health Europe	Network of mental health users, professionals, service providers across Europe
Nature	Scientific publication
Healthonline	Online health magazine/resource
Somerset council	Local government council
MARCH	Research network
Mental health foundation	Mental health charity

Table 2 provides a summary of the results, which are described in further detail below.

**Table 2:** Content of COVID-19 mental health focused online resources.

**Strategy**	**Sub-strategy**	**Number of resources (%)**
Normalizes		30 (77)
Social support	Stay/get connected socially	34 (87)
	Help other people	24 (62)
Physical self-care	General advice including diet, smoking, alcohol	23 (59)
	Exercise	23 (59)
	Sleep	13 (33)
Media	Access trustworthy information	23 (59)
	Manage/limit media intake	23 (59)
Activity	Routine	22 (56)
	Do activities/stay busy	13 (33)
	Specifically do useful things	7 (18)
	Specifically do what you enjoy	16 (41)
	Specifically learn new things/keep mind active	11 (28)
	Specifically do creative things	8 (21)
	Specifically be outside/in contact with nature	10 (26)
	Set goals	6 (15)
Cognitive strategies	Mindfulness/focus on present	17 (44)
	Focus on what you can control	14 (36)
	Cognitive reframing	18 (46)
	Problem solving	3 (8)
Relaxation/managing arousal		16 (41)
Emotional expression		12 (31)
Relationship management/communication strategies		7 (18)
Attending to physical environment		4 (10)

### Framing of the Situation, Impact, and Resource

The COVID-19 pandemic was mostly referred to as “COVID-19 outbreak” or simply as “COVID-19” or “coronavirus.” Almost all resources specifically named the situation, with the language of outbreak, virus name, pandemic or epidemic used. Just four resources did not use this language, instead referring to “lockdown,” “crisis,” or “social isolation.” Potentially stigmatizing language of “Wuhan virus” or “Chinese virus” were not seen.

The impact of the situation on psychological wellbeing and mental health typically included significant attempts to normalize. Resources described the situation as having an impact on everyone, emphasizing universality. Worry and stress were described as “natural,” “normal” and “understandable.” Many described that everyone would react differently. Some described examples of specific people's experiences, to exemplify potential reactions. Of the 39 inspected resources, nine did not offer normalization of psychological distress. Some of these were very brief resources, however four were resources, longer than a list of tips, from mental health charities. Most resources did not use diagnostic labels to describe people's experiences, and offer normalization of distress. Where diagnostic categories were used and where symptoms or difficulties were described, the focus was on anxiety.

Whilst resources described a range of potential emotional and psychological impacts, the majority focused on anxiety and its management, focusing on “worry,” “stress,” “anxiety,” “fear” and “uncertainty”.

The resources were published by a range of organizations, as show in Table 1. The results reflect to some extent the geographical location of the authors, and cover international, national, and local sources, from governments, charities, businesses, and individuals. They are mostly lists of instructions, sometimes with text preceding these instructions. The intended aims or claims of what the resources could offer were commonly to “look after,” “help,” “support,” “take care,” “manage” and “protect.” No resource made claims to remove distress or treat.

### Content of Resources

The resources offered a range of strategies, detailed in Table 2. The majority of resources offered a list of tips or strategies. The resources then were frequently very brief, offering little detail on how to implement the suggested tips or strategies.

It was rare to see the likelihood of achieving these acknowledged, with just one resource explicitly suggesting “cut yourself some slack.” Managing expectations of oneself was rarely acknowledged. The majority of advice did not acknowledge the other challenges that people may be facing, including lack of time owing to caring responsibilities, ongoing work expectations and challenges, potential ill-health, financial stressors, and difficulties in managing how to obtain everyday items such as food.

Social support was most commonly suggested. Self-care, management of media usage, maintenance of activities, and use of cognitive strategies were all advised. Several resources included content on focusing on what one can control as a cognitive strategy. Arousal reduction and emotional expression were also offered. Less commonly offered were advice relating to managing personal relationships and maintaining or altering one's physical environment. Within the cognitive focused advice, several resources offer prompts to consider the positives or solve problems, without offering further guidance on how this can be achieved. Some suggested the situation be reframed as an opportunity. An emphasis on kindness and gratitude was also seen.

### Comparison of Resource Coverage to Mental Health Impact

Data from the ONS observed an increase in depression in the general population from pre-pandemic levels of around 10 to 19% in June and November 2020, and 21% in January-March 2021 (20). These data relate to those aged 16 or more. Interestingly, depression was most common at 34% in the 16–29 year old age range (compared to just 10% in people aged at least 70 years). This age group are of course highly internet literate. For anxiety, in March-April 2020, at the very start of the pandemic in the UK and during a period of extreme uncertainty, 49.6% of people reported high anxiety (21). The mean score of the anxiety measure was 5.2/10, up from 3.0/10 at the end of 2019. Data from July-August 2021 show on ongoing higher than pre-pandemic level of depression, being 17% (compared to pre-pandemic levels of 10%) (22).

## Discussion

Analysis examined the framing and content of a variety of online resources aiming to support psychological wellbeing. The epidemic was referred to without using stigmatizing language for either the virus or the impact on mental wellbeing. Normalizing and contextualizing distress during a distressing situation is vital, as it can offer a sense of universality and not stigmatize what is understandable during this time of threat. Resources were found to be stating aims to help support mental wellbeing, which is an appropriate aim as these mass, online resources are not to replace therapy or interventions to treat mental health problems.

Overall the content of interventions could be broadly construed as including elements of cognitive-behavioral therapies, including elements of mindfulness and acceptance commitment approaches, such as thought defusion (27). The focus on maintaining activity and social contact address likely stressors of the situation. Practical strategies are frequently offered. These strategies can address both low mood and anxiety related difficulties as they form elements of behavioral activation (28). Cognitive intervention is overall less frequently offered, and again tends to focus on anxious thinking, however could be equally well applied to depressive rumination, for example techniques of reframing and defusing from thoughts may be useful for both types of difficulties (29). Control is a theme in nearly half the resources, typically with advice to focus on what you can control. This is highly relevant as appropriate control beliefs are linked to reduced anxiety (16). This is particularly important messaging to avoid fatalism, which may reduce protective health behaviors of handwashing and social distancing (15).

The data concerning the impact of the pandemic show a large increase in both depression and anxiety. A clear recommendation results from comparing this to the content of the resources. The majority of resources focused on anxiety related symptoms and strategies. It is vital ensure impact on depressive symptoms is also acknowledged, given preliminary findings of increased depression (8).

A number of additional recommendations arise from the findings. First, most resources were didactic information giving. These could usefully be augmented with guided activities, to increase engagement with the resources' advice. This could include links to activities to support people to problem solve, cognitively reframe, set goals and so on. Most people are likely to be able to spontaneously do these activities, however some may require further support. Second, the pandemic events are outside of personal control, potentially leading feelings of helplessness, which are linked to low mood and depression (30). More attention should be given in resources to reducing rumination, replacing both anxious and low-mood related thoughts with alternatives or defusing from thoughts. Third, the resources' content could potentially feel overwhelming itself: a single parent, struggling to manage a toddler at home and continue to work, experiencing financial difficulties and stress when trying to acquire food shopping may find a list suggesting great social contact and doing creative activities somewhat at odds with their personal experience. Noting the context is vital and recognizing this may be having a very significant impact may help some to feel more understood and the resources to feel more relevant. Fourth, and related to the previous recommendation, it may be important to include guidance on adapting ones' expectations of oneself. The current challenges may activate core beliefs about being not good enough, which may be further triggered by lists of activities to do. This could be simply acknowledging a need to shift one's expectations from normal level of achievement or activity to abnormal ones, given the abnormal situation. Fifth, resources could continue to emphasis the rationale of the restrictions to movement, emphasizing the shared experience; social cohesion and resilience; and collective responsibility to act, in the collective good (31, 32).

Finally, two areas were commonly omitted. First, the importance of managing personal relationships was rarely mentioned. It is important to note that loneliness was observed in 27% of a UK sample in March-April 2020 (33). Addressing the maintenance of relationships during times of protective measures that restrict social interaction is then vital. In addition to the significant risk of increased domestic violence, the stress of confinement may affect many relationships within the home. Raising this issue and inclusion of basic guidance on communication skills may be beneficial (34). Second, greater awareness raising of the potential impact on mental wellbeing of home environment may be beneficial, particularly as this is an area over which many people will have some control (35).

### Limitations

This study is limited by its sampling. Only resources written in English were included, limiting its coverage and generalisability, however resources from a range of sources were included. The limitation to resources written in English does also limit the underlying cultural assumptions and approaches offered. Only resources relating to a general population were included, advice for health-workers and children must also be made available to the highest standard. It is not possible to review the resources against existing evidence standards, as no such standards exist; rather we sought to summarize the content, consider its empirical basis, and highlight areas of omission.

## Conclusion

In conclusion, the resources relating to public mental health addressed important topics. Supportive, comprehensive, empirically grounded resources can help maintain mental wellbeing for many, thus reducing future impact on mental health services, allowing them to focus on those with existing difficulties and high levels of distress. Such resources must address not only anxiety, but also depression. Given the high risk of future pandemics, the recommendations may inform future mass mental health support interventions.

## Data Availability Statement

The original contributions presented in the study are included in the article/supplementary material, further inquiries can be directed to the corresponding author/s.

## Author Contributions

FM conceived of the study, coded data, and co-drafted the manuscript. TO gave methodological advice, coded data, co-drafted, and edited the manuscript. All authors contributed to the article and approved the submitted version.

## Conflict of Interest

The authors declare that the research was conducted in the absence of any commercial or financial relationships that could be construed as a potential conflict of interest.

## Publisher's Note

All claims expressed in this article are solely those of the authors and do not necessarily represent those of their affiliated organizations, or those of the publisher, the editors and the reviewers. Any product that may be evaluated in this article, or claim that may be made by its manufacturer, is not guaranteed or endorsed by the publisher.

## References

[1] Brooks SK Webster RK Smith LE Woodland L Wessely S Greenberg N. The psychological impact of quarantine and how to reduce it: rapid review of the evidence. The Lancet. (2020) 395:912–20. 10.1016/S0140-6736(20)30460-8 32112714 PMC7158942

[2] Chew QH Wei KC Vasoo S Chua HC Sim K. Narrative synthesis of psychological and coping responses towards emerging infectious disease outbreaks in the general population: practical considerations for the COVID-19 pandemic. Singapore Med J. (2020) 20:22. 10.11622/smedj.2020046 32241071 PMC7926608

[3] Chung J Yeung W. Staff mental health self-assessment during the COVID-19 outbreak. East Asian Arch Psychiatry. (2020) 20:12. 10.12809/eaap2014 32229646

[4] Lai J Ma S Wang Y Cai Z Hu J Wei N. Factors associated with mental health outcomes among health care workers exposed to coronavirus disease 2019. JAMA Netw Open. (2020) 3:e203976–e20. 10.1001/jamanetworkopen.2020.3976 32202646 PMC7090843

[5] Li S Wang Y Xue J Zhao N Zhu T. The impact of COVID-19 epidemic declaration on psychological consequences: a study on active weibo users. Int J Environ Res Public Health. (2020) 17:2032. 10.3390/ijerph17062032 32204411 PMC7143846

[6] Tan BYQ Chew NWS Lee GKH Jing M Goh Y Yeo LLL. Psychological impact of the COVID-19 pandemic on health care workers in Singapore. Ann Intern Med. (2020) 12:84. 10.7326/M20-1083 32251513 PMC7143149

[7] Wang C Pan R Wan X Tan Y Xu L Ho CS. Immediate psychological responses and associated factors during the initial stage of the 2019 coronavirus disease (COVID-19) epidemic among the general population in China. Int J Environ Res Public Health. (2020) 17:1729. 10.3390/ijerph17051729 32155789 PMC7084952

[8] Bentall RP, Gibson-Miller, J, Levita, L, Hartmann, T, Martinez, A, Stocks, T,. Initial Research Findings on COVID-19 Mental Health in the UK, COVID-19 Psychological Research Consortium (C19PRC). (2020). Available online at: https://drive.google.com/file/d/1A95KvikwK32ZAX387nGPNBCnoFktdumm/view?usp=sharing (accessed March 31, 2020).

[9] Zhang S Wang Y Rauch A Wei Fe. Health, Distress and Life Satisfaction of People in China One Month into the COVID-19 Outbreak. Lancet. (2020) 20:5216. 10.2139/ssrn.3555216 PMC7146665 32283450

[10] Bao Y Sun Y Meng S Shi J Lu L. 2019-nCoV epidemic: address mental health care to empower society. The Lancet. (2020) 395:e37–e38. 10.1016/S0140-6736(20)30309-3 32043982 PMC7133594

[11] Liu S Yang L Zhang C Xiang Y-T Liu Z Hu S. Online mental health services in China during the COVID-19 outbreak. The Lancet Psychiatry. (2020) 7:e17–e18. 10.1016/S2215-0366(20)30077-8 32085841 PMC7129099

[12] Torous J Jän Myrick K Rauseo-Ricupero N Firth J. Digital mental health and COVID-19: using technology today to accelerate the curve on access and quality Tomorrow. JMIR mental health. (2020) 7:e1. 10.2196/18848 32213476 PMC7101061

[13] Ho CS Chee CY Ho RC. Mental health strategies to combat the psychological impact of COVID-19 beyond paranoia and panic. Ann Acad Med Singapore. (2020) 49:1–3. 10.47102/annals-acadmedsg.202043 32200399

[14] Smith J Q&A: Future pandemics are inevitable but we can reduce the risk. (2021). Available online at: https://ec.europa.eu/research-and-innovation/en/horizon-magazine/qa-future-pandemics-are-inevitable-we-can-reduce-risk: Horizon: The EU Research and Innovation Magazine (accessed December 11, 2021).

[15] Ramondt S Ramírez AS. Fatalism and exposure to health information from the media: examining the evidence for causal influence. Ann Int Commun Assoc. (2017) 41:298–320. 10.1080/23808985.2017.1387502 34307882 PMC8297407

[16] Slovic P Peters E. Risk perception and affect. Curr Direct Psychol Sci. (2006) 15:322–5. 10.1111/j.1467-8721.2006.00461.x

[17] DiMatteo M Lepper H Croghan T. Depression is a risk factor for noncompliance with medical treatment: meta-analysis of the effects of anxiety and depression on patient adherence. Archiv Intern Med. (2000) 160:2101–7. 10.1001/archinte.160.14.2101 10904452

[18] Kroenke K Spitzer RL Williams JB. The PHQ-9: validity of a brief depression severity measure. J Gen Intern Med. (2001) 16:606–13. 10.1046/j.1525-1497.2001.016009606.x 11556941 PMC1495268

[19] Spitzer RL Kroenke K Williams JBW Lowe B. A brief measure for assessing generalized anxiety disorder. Arch Intern Med. (2006) 166:1092–7. 10.1001/archinte.166.10.1092 16717171

[20] Office for National Statistics Coronavirus and depression in adults Great Britain: January to March 2021 Coronavirus and depression in adults Great Britain Office for National Statistics. (2021)

[21] Office for National Statistics. Coronavirus and Anxiety, Great Britain: 3 April 2020 to 10 May 2020. (2020). Available online at: https://www.ons.gov.uk/peoplepopulationandcommunity/wellbeing/articles/coronavirusandanxietygreatbritain/3april2020to10may (accessed January 122022).

[22] Office for National Statistics Coronavirus (COVID-19) latest insights: Wellbeing A.o.J. (2022). Available online at: https://www.ons.gov.uk/peoplepopulationandcommunity/healthandsocialcare/conditionsanddiseases/articles/coronaviruscovid19latestinsights/wellbeing#mental-health

[23] Mayring P. “Qualitative content analysis,” in Editores U. Flick, E. von Kardoff, I. Steinke, A Companion to Qualitative Research. (2004) Sage: London. p. 266–269.

[24] Rowlands T Waddell N McKenna B. Are we there yet? a technique to determine theoretical saturation. J Comput Inform Syst. (2016) 56:40–7. 10.1080/08874417.2015.11645799

[25] Glaser BG Strauss AL. Discovery of Grounded Theory: Strategies for Qualitative Research. London: Routledge. (2017).

[26] Cohen J. Weighted kappa: nominal scale agreement with provision for scaled disagreement or partial credit. Psychol Bull. (1968) 70:213–20. 10.1037/h0026256 19673146

[27] Hayes SC Strosahl KD Wilson K Acceptance G. Commitment Therapy: The Process and Practice of Mindful Change. New York, NY: Guilford Press (2012).

[28] Richards DA Ekers D McMillan D Taylor RS Byford S Warren FC. Cost and outcome of behavioural activation versus cognitive behavioural therapy for depression (COBRA): a randomised, controlled, non-inferiority trial. The Lancet. (2016) 388:871–80. 10.1016/S0140-6736(16)31140-0 27461440 PMC5007415

[29] Hayes SC Hofmann SG. Process-based CBT: The science and core clinical competencies of cognitive behavioral therapy. London: New Harbinger Publications (2018).

[30] Levin J. “Mental health assistance to families and communities in the aftermath of an outbreak,” In: Editor D. Huremović Psychiatry of Pandemics: A Mental Health Response to Infection Outbreak. (2019) Cham: Springer International Publishing, p. 143-151.

[31] Reicher S Drury J. Don't personalise, collectivise! The Psychologist. (2020). Available online at: https://thepsychologist.bps.org.uk/dont-personalise-collectivise (accsessed April 2, 2020).

[32] Townshend I Awosoga O Kulig J Fan H. Social cohesion and resilience across communities that have experienced a disaster. Nat Hazards. (2015) 76:913–38. 10.1007/s11069-014-1526-4

[33] Groarke JM Berry E Graham-Wisener L McKenna-Plumley PE McGlinchey E Armour C. Loneliness in the UK during the COVID-19 pandemic: Cross-sectional results from the COVID-19 psychological wellbeing study. PLOS ONE. (2020) 15:e023. 10.1371/journal.pone.0239698 32970764 PMC7513993

[34] Askari M Mohd Noah S Aishah Hassan S Baba M. Comparison of the effects of communication and conflict resolution skills training on mental health. Int J Psychol Stud. (2013) 13:84. 10.5539/ijps.v5n1p91

[35] Saxbe DE Repetti R. No place like home: home tours correlate with daily patterns of mood and cortisol. Personal Soc Psychol Bull. (2010) 36:71–81. 10.1177/0146167209352864 19934011

